# Supplementary material for: Engineering electro-crystallization orientation and surface activation in wide-temperature zinc ion supercapacitors
Source: Nat Commun. 2025 Apr 16;16:3597. doi: 10.1038/s41467-025-58857-5 (PMC12000396; doi:10.1038/s41467-025-58857-5)
Supplement: Supplementary file 1 — Supplementary Information [file 41467_2025_58857_MOESM1_ESM.pdf]

Engineering Electro-crystallization Orientation and Surface Activation in Wide-Temperature Zinc Ion Supercapacitors

Lulu Yao[1], Nandu Koripally[2], Chanho Shin[1], Anthony Mu,[3] Zheng Chen,[1,3,4] Kaiping Wang[1], Tse Nga Ng[1,2,4] *

[1]Program of Materials Science and Engineering, University of California, San Diego, La Jolla, CA 92093, United States of America.
[2]Department of Electrical and Computer Engineering, University of California, San Diego, La Jolla, CA 92093, United States of America.
[3]Aiiso Yufeng Li Family Department of Chemical and Nano Engineering, University of California, San Diego, La Jolla, CA 92093, United States of America.
[4]Sustainable Power and Energy Center, University of California San Diego, La Jolla, CA 92093, United States of America.
*Corresponding email: tnn046@ucsd.edu

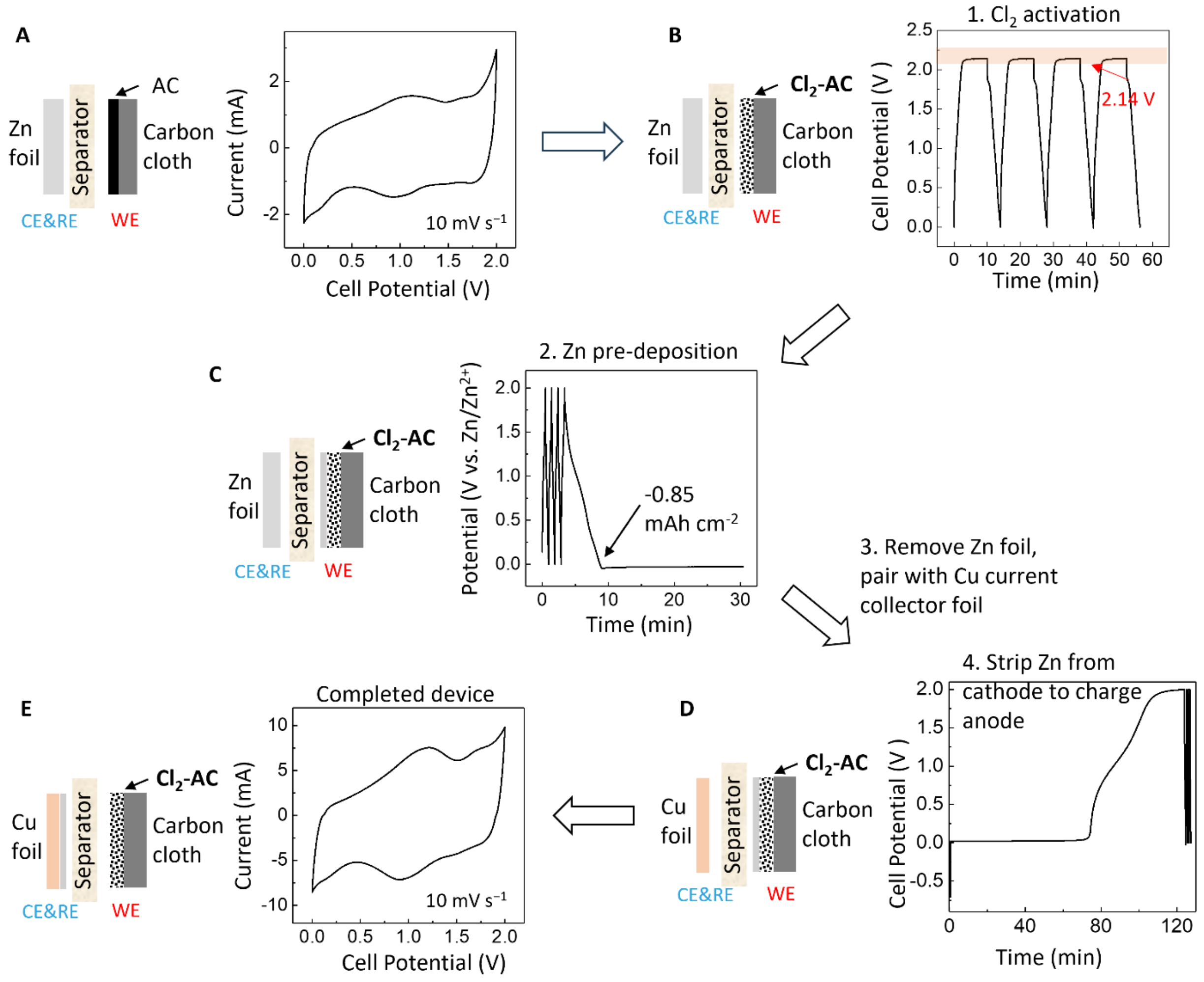

Figure S1. (A) Cyclic voltammetry (CV) curve of typical AC//Zn foil supercapacitor. (B) Activation of AC cathode inside 15 M $ZnCl_2$ electrolyte. (C) The deposition of Zn from a Zn foil to the cathode by applying a negative current to the working electrode, then monitoring the voltage change. (D) After replacing the Zn foil with a Cu current collector foil, Zn on the cathode side was stripped and then plated on the anode side. (E) The CV curve of the completed anode-free device.

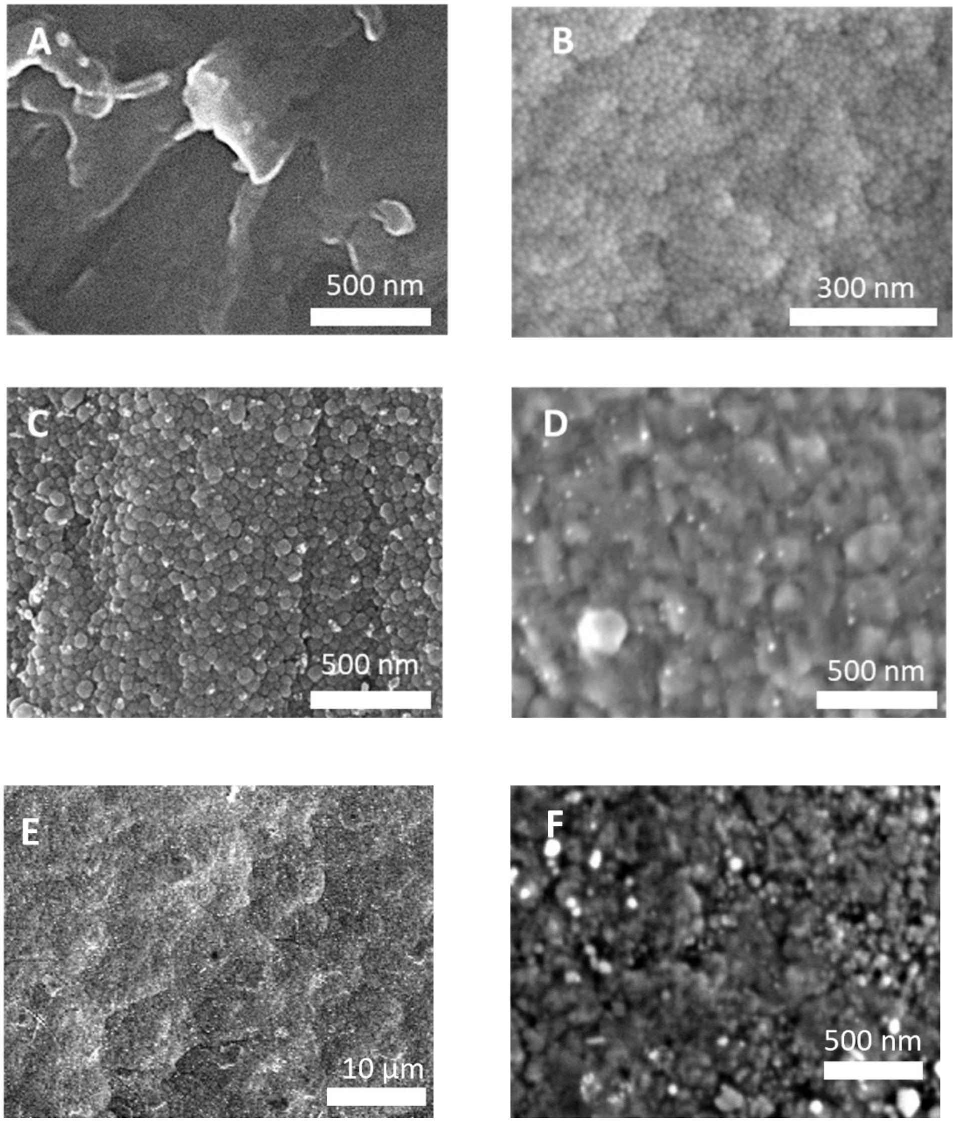

Figure S2. Scanning electron microscopy of (A) the original Cu foil surface. (B, C, and D) Copper foil surface after sputtering copper nanoparticles at different RF power and time duration. (B) 6 min at 100 W power. (C) 3 min at 100 W power. (D) 3 min at 200 W power. The particle size of copper nanoparticles decreased with smaller sputtering power, where the aggregates at 100W have a diameter <20 nm and at 200 W have a diameter ~200 nm. (E, F) Surface morphology of the anode at 0% state-of-charge after 1000 redox cycles. Cu nanoparticles stayed intact on the anode current collector.

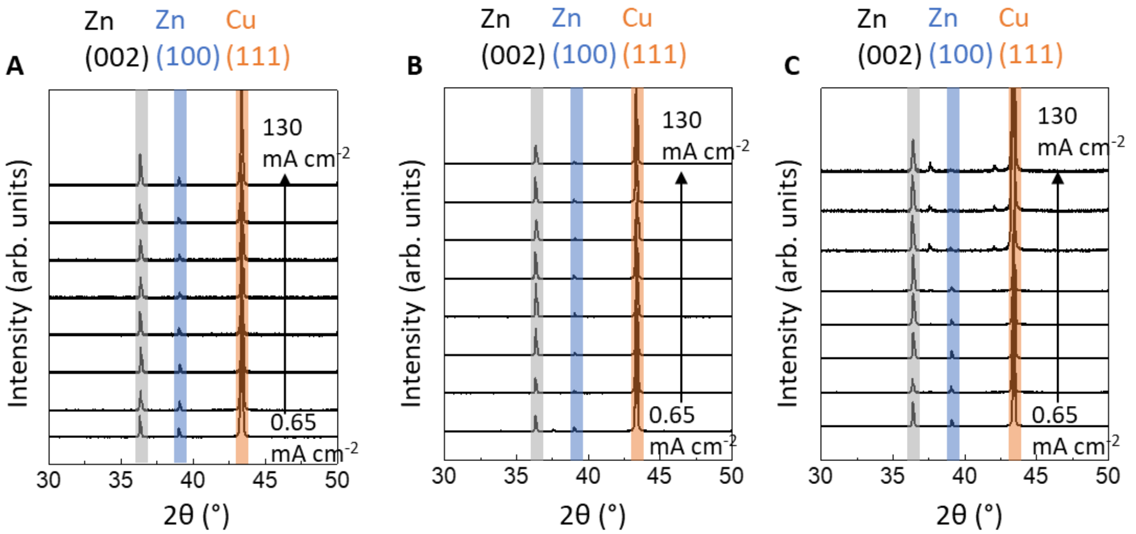

Figure S3. X-ray diffraction results of Zn plating on (A) a bare Cu foil and (B) 150 W sputtered CuNPs on Cu foil, and (C) 200 W sputtered CuNPs on Cu foil. The electrodeposition current densities are 0.65, 1.3, 6.5, 13, 26, 52, 104, 130 mA cm^{-2}.

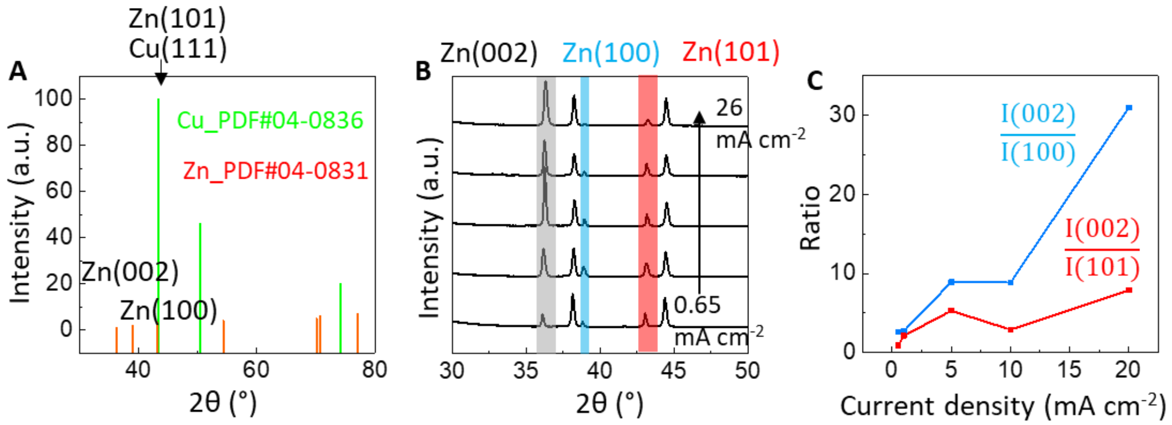

Figure S4. X-ray diffraction peaks of (A) reference Cu (PDF#04-0836) and Zn foils (PDF#04-0831). (B) Zn electrodeposition at different current density (0.65, 1.3, 6.5, 13, 26 mA cm^{-2}) on a graphite surface with copper nanoparticles formed by RF sputtering power at 150 W for 3 min. (C) Ratio of x-ray peaks versus electrodeposition current density in part (b).

To analyze the crystal facets in the electrodeposited Zn layer, we have used different current collectors, such as graphite foil or Al foil, to avoid the peak overlap of copper foil. In Supplemental Figure S3B, graphite foil was used as the current collector, with its surface modified by sputtering a layer of copper nanoparticles. We observed that the peak intensity ratios $I(002)/I(100)$ and $I(002)/I(101)$ followed the same trend and either would work well for monitoring the zinc facets. In the main text, $I(002)/I(100)$ was employed to conducted the analysis on the plating/stripping process

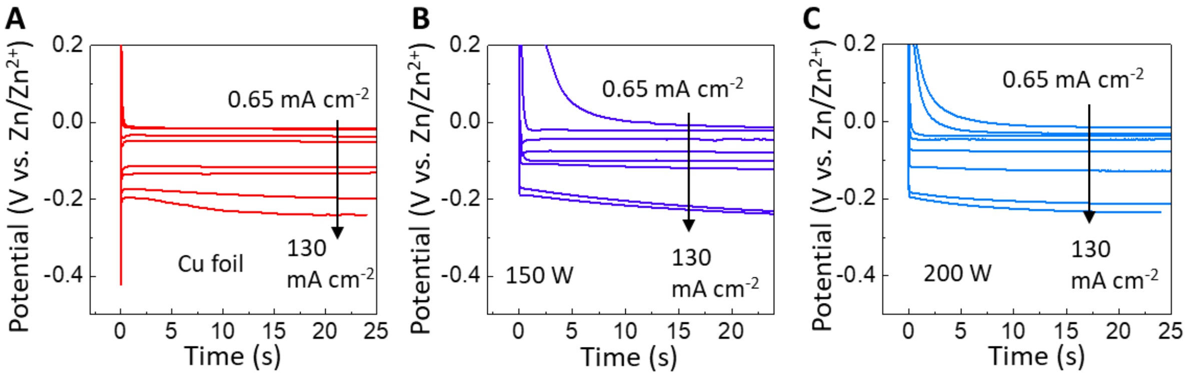

Figure S5. Voltage profiles of Zn deposition on (A) a bare Cu foil and (B) 150 W sputtered CuNPs-Cu foil (CuNPs diameter ~50 nm), and (C) 200 W sputtered CuNPs-Cu (CuNPs diameter ~200 nm), at different current densities of 0.65, 1.3, 6.5, 13, 26, 52, 104, 130 mA cm^{-2}.

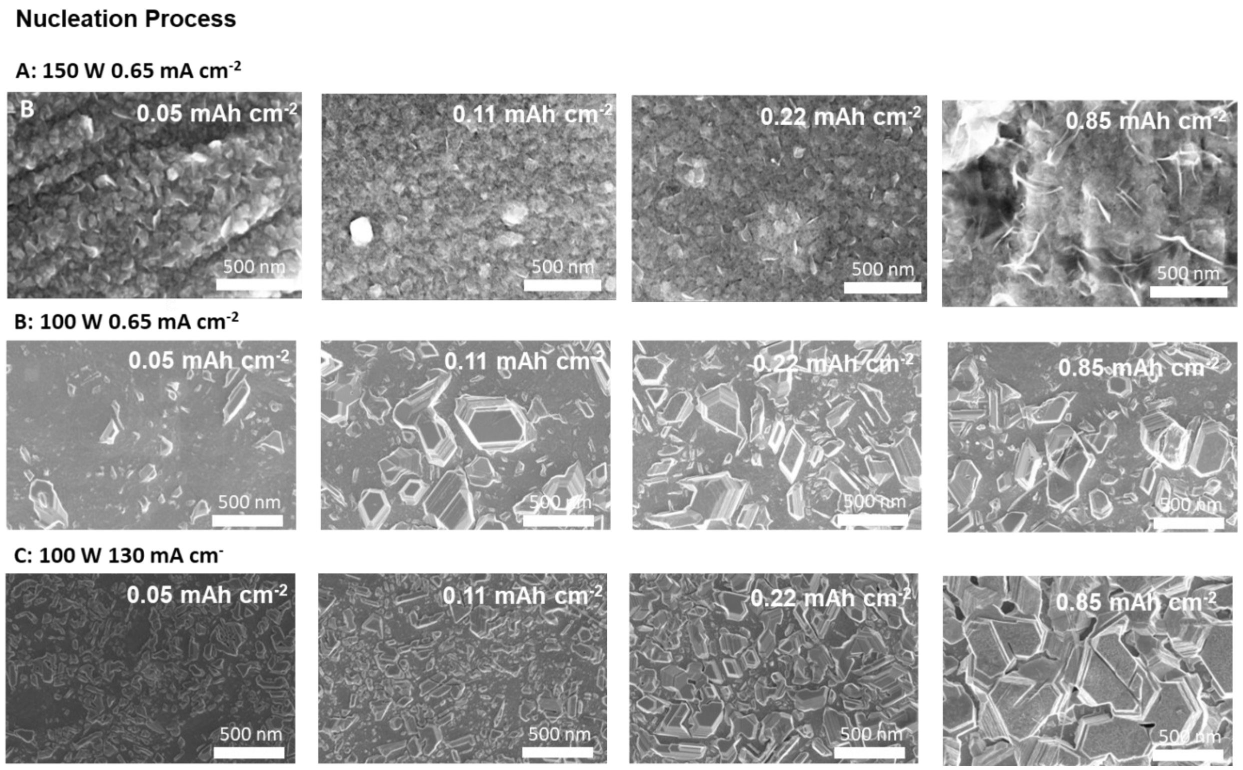

Figure S6. Scanning electron microscopy images of the Zn layer on (A) 150 W sputtered CuNPs-Cu, with Zn deposited at a current density of 0.65 mA cm^{-2}. (B) 100 W sputtered CuNPs-Cu, with Zn deposited at 0.65 mA cm^{-2} current density. (C) 100 W sputtered CuNPs-Cu, with Zn deposited at 130 mA cm^{-2} current density. These three conditions were highlighted because they led to different *I*(002)/*I*(100) ratios, as reflected in the different grain morphologies in the images.

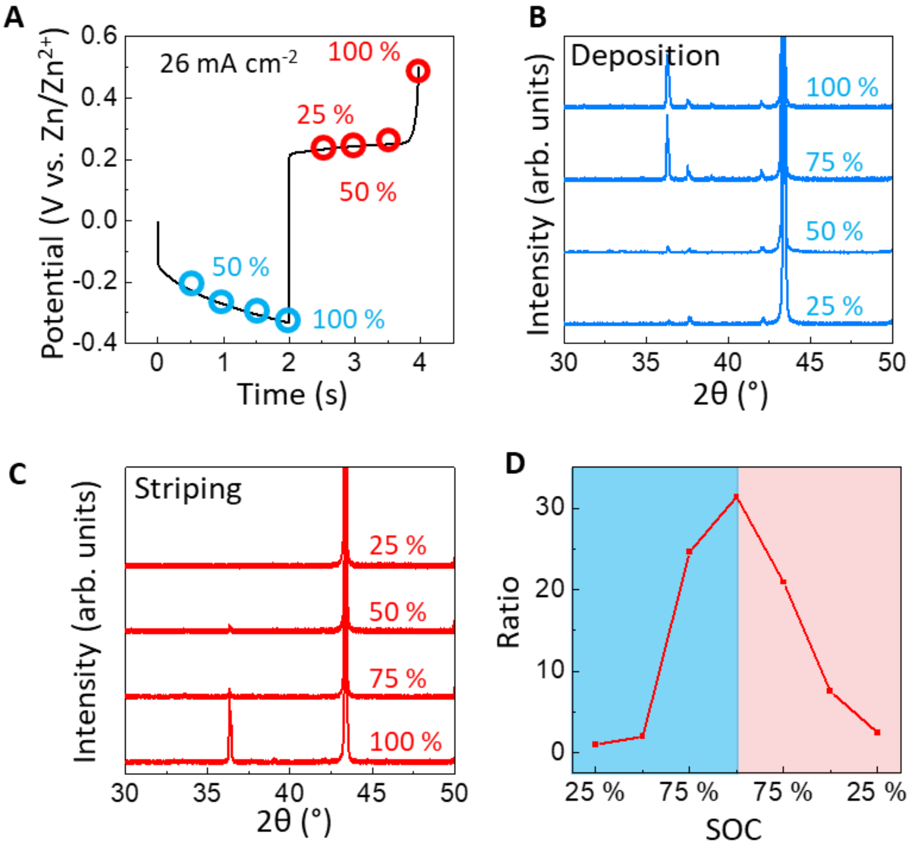

Figure S7. (A) Voltage profile of a typical charging/discharge process of the Zn anode where the current collector surface was modified with CuNPs. (B) X-ray diffraction results of Zn deposition at various state-of-charge increasing from 25% to 100%, and (C) Zn stripping from 100% to 25%. (D) The peak intensity ratio of *I*(002)/*I*(100) as a function of the state of charge.

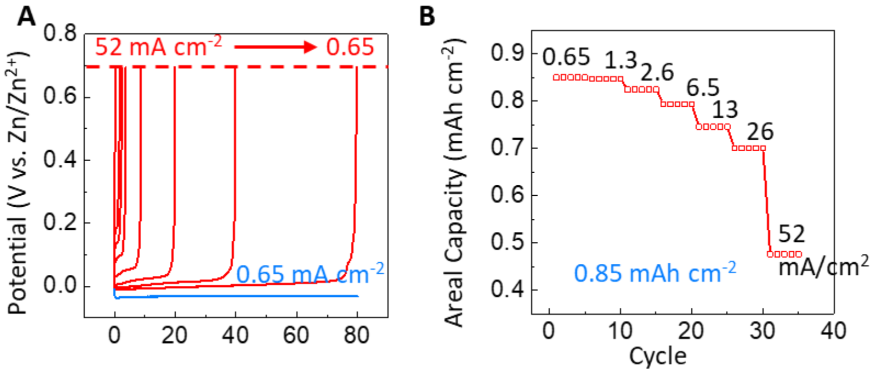

Figure S8. (A) Voltage profile of discharging process (red) of the Zn anode at different current densities (0.65, 1.3, 2.6, 6.5, 13, 26, 52 mA cm^{-2}). The blue curve is for the charging process. (B) The corresponding areal capacity changes with current density.

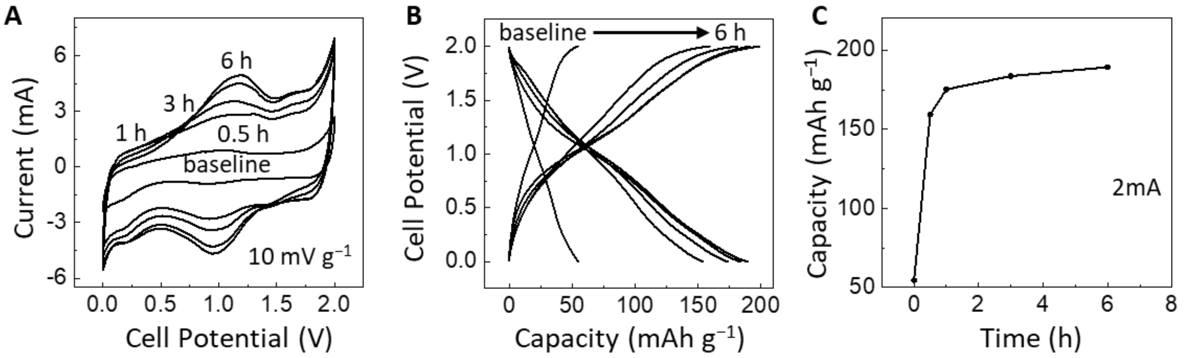

Figure S9. (A) Cyclic voltammetry curves and (B) Capacity-voltage profiles of Cl_2 gas activated active carbon measured in 15 M $ZnCl_2$ electrolyte. The activation periods were 0 h, 0.5 h, 1 h, 3 h, and 6 h. (C) Capacity change as a function of activation time.

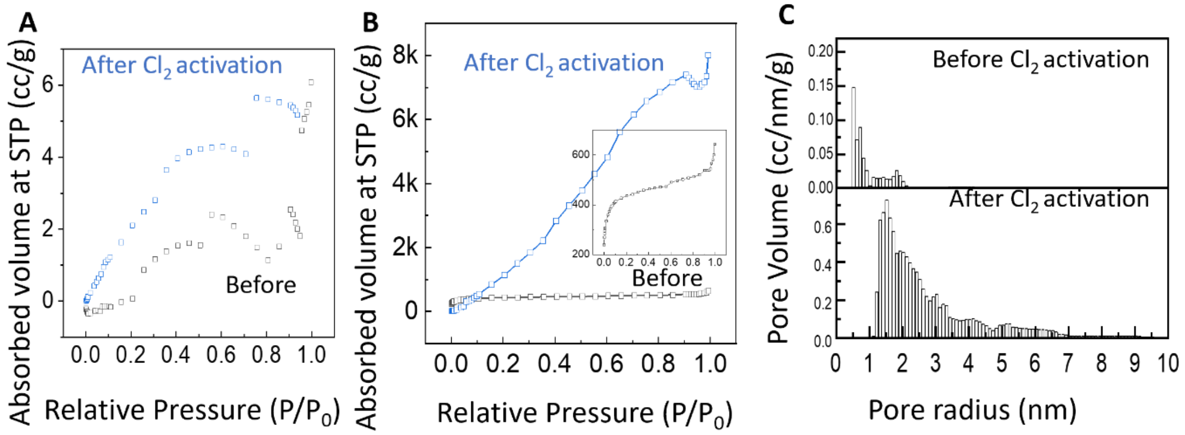

Figure S10. Brunauer-Emmett-Teller (BET) surface analysis. Before and after Cl_2 activation of (A) a carbon cloth (CC) substrate; (B) activated carbon (AC) on CC substrate. (C, D) Histograms of pore radius distribution.

Table S1. Sample surface area and porosity from BET measurements. STP: Standard temperature and pressure.

	Surface area (m^2/g)	Total pore volume at STP (cc/g)
Carbon cloth (CC)	6	Not applicable
CC after Cl_2 activation	12	Not applicable
AC on CC	1670	1.08
AC on CC after Cl_2 activation	5750	13.86

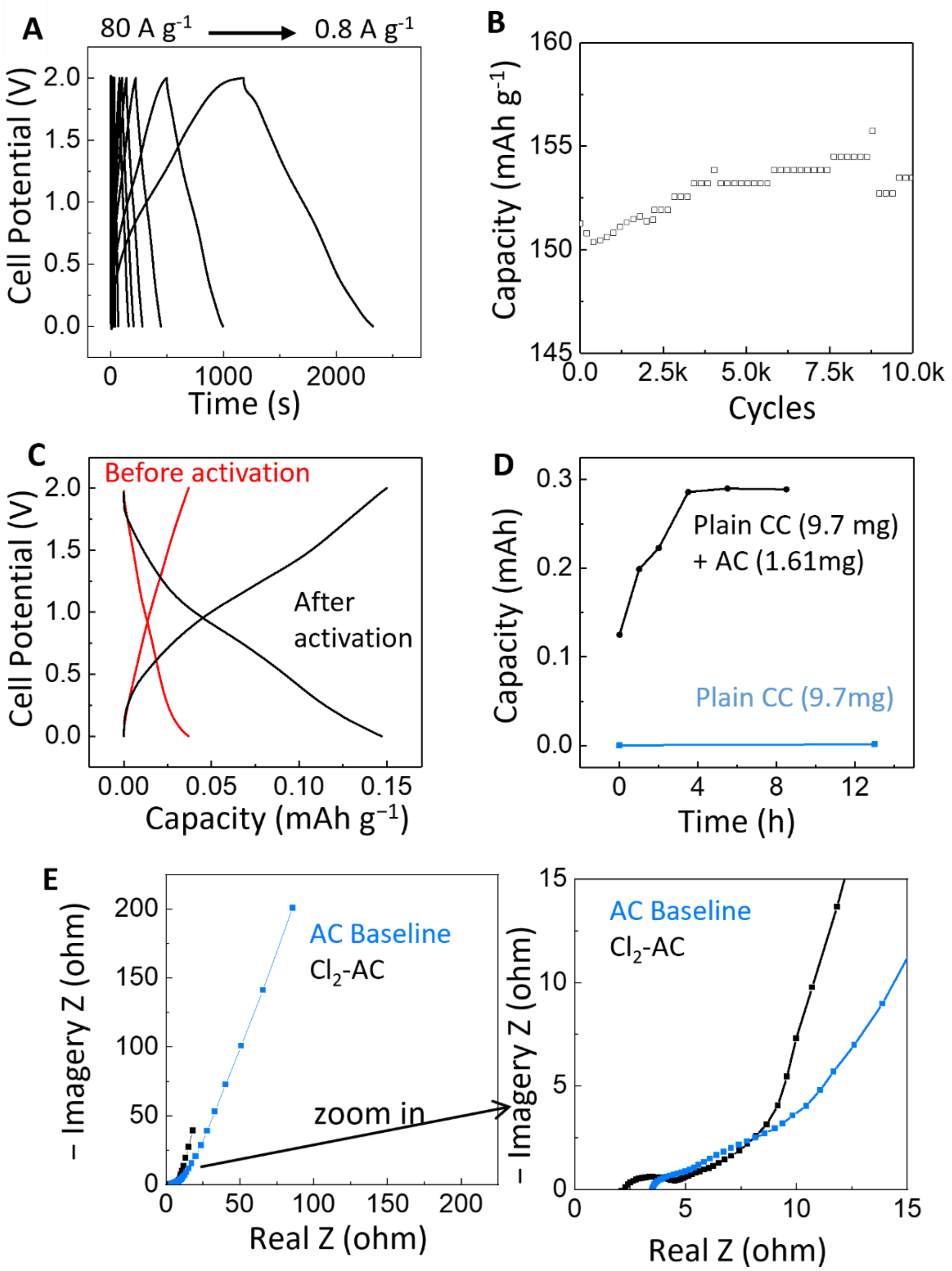

Figure S11. (A) Voltage profiles of the Cl_2-activated cathode at various charge/discharge current ranging from 0.8 A g^{-1} to 80 A g^{-1}, corresponding to Fig. 4E in the main tex. (B) Charging/discharging stability test of Cl_2 activated AC cathode. The voltage was between 0 and 2 V and the current density was 80 A g^{-1}. (C) Capacity-voltage profiles of the plain carbon cloth (CC) and Cl_2-activated CC (activated for 16 h), measured at a current density of 2 mA. (D) Capacity versus activation time, on samples of plain CC and CC with active carbon. (E) Imaginary impedance versus real impedance of AC before and after Cl_2 activation, measured at 1 V versus a Zn/Zn^{2+} reference.

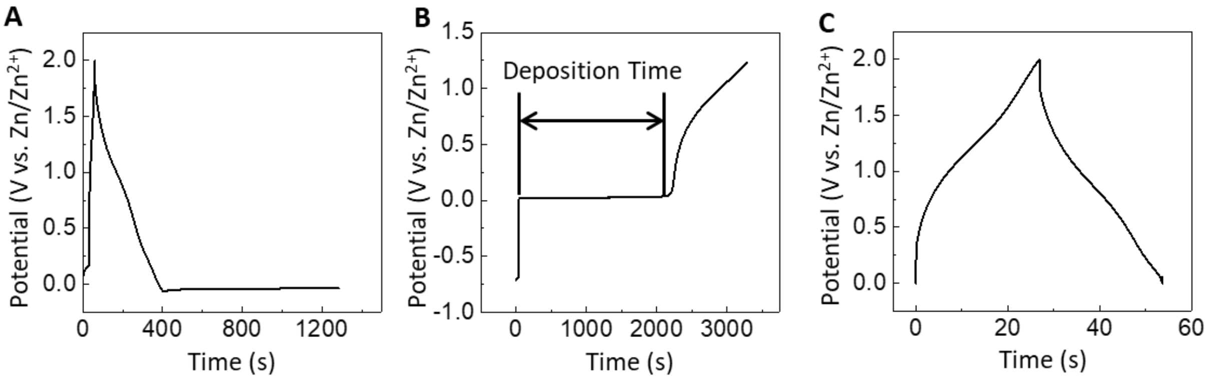

Figure S12. The calculation method of n/p ratio for anode free devices. (A) The voltage profile of Zn deposition on AC cathode. Deposition current density: 2.6 mA cm^{-2}. (B) The deposition of Zn from AC cathode to CuNPs-Cu substrate. Deposition current density: 0.65 mA cm^{-2}. (C) The galvanostatic charge-discharge (GCD) curve of the device at an applied current density of 26 mA cm^{-2}.

The mass of electroplated zinc and its utilization ratio are calculated as follows:

1 mole electrons carry 96500 coulombs

Capacity of anode = Current × time = 0.65 mA cm^{-2} × 2142 s (from part B) = 1392.3 mA s cm^{-2}

mole of Zn = Current × time/96500 = 0.00065 A cm^{-2} × 2142 s / 96500 / (2 electrons per Zn^{2+}) = 7.21 μmol cm^{-2}

Loading of Zn = 7.21 μmol cm^{-2} × 65.38 g/mol = 0.472 mg

Capacity of cathode = 26 mA cm^{-2} × 26.7 s = 694 mA s cm^{-2} (from part C)

$$\text{Utilization ratio} = \frac{Capacity\ of\ Cathode}{Capacity\ of\ Anode} = \frac{694}{1392.3} = 49.9\ \%$$

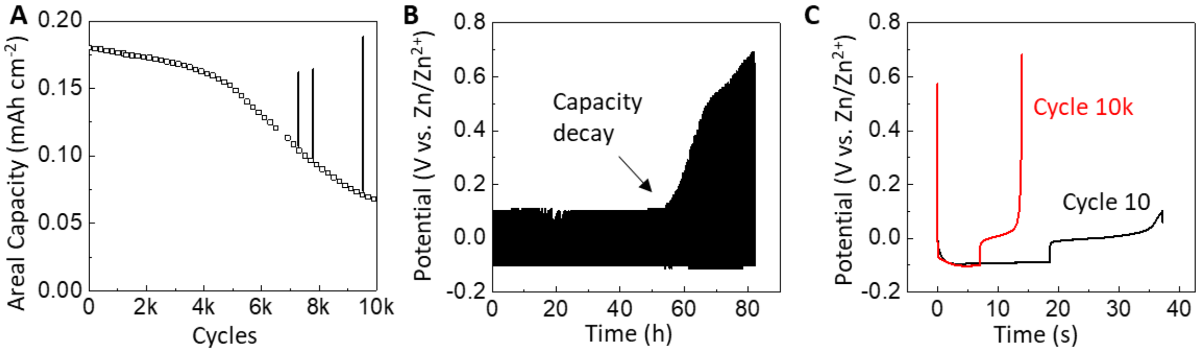

Figure S13. (A) Cycling performance of device using AC||bare Cu foil, with a n/p ratio of 4. (B) Cell potential versus time at the anode side. (C) The charging/discharging cycle of the anode with a bare Cu foil at cycle 10 and cycle 10,000.

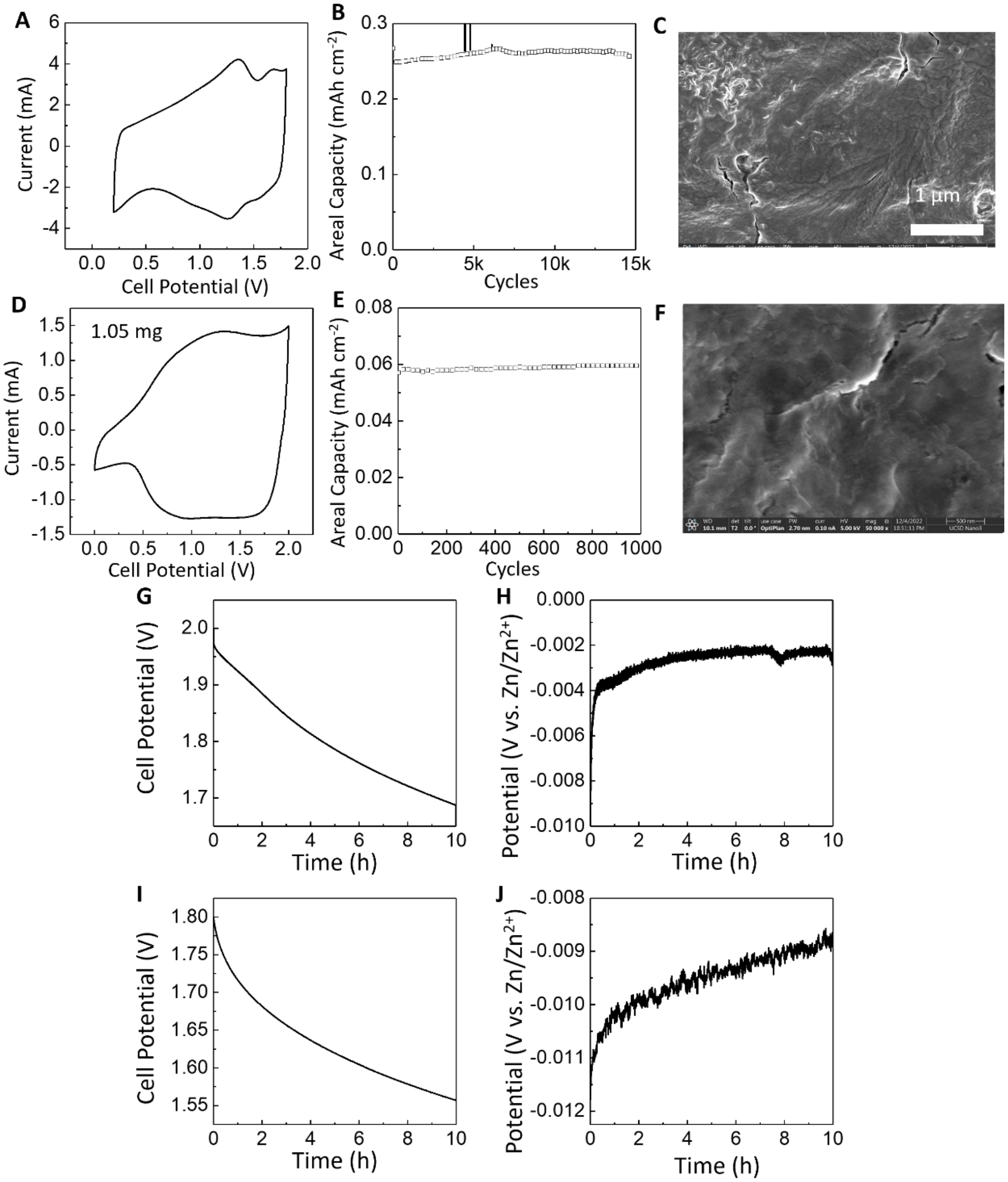

Figure S14. (A) Cyclic voltammetry (CV) and (B) cycling performance of a device (N/P=4) with 2 M $ZnSO_4$ in deionized water as the electrolyte. (C) Surface morphology of the anode after 1,000 full charging/discharging cycles in part B. (D) CV curve and (E) cycling performance of a device (N/P=4) with 2 M Zinc bis(trifluoromethylsulfonyl)imide in propylene carbonate as the electrolyte. (F) Surface morphology of the anode after 1,000 full charging/discharging cycles in part E. (G) Self-discharge voltage drop of a device with 15 M $ZnCl_2$ electrolyte. (H) The potential change of Zn on CuNPs-Cu anode during the self-discharge process. (I) Self-discharge performance of a device with 2 M $ZnSO_4$ electrolyte. (J) The potential change of Zn on CuNPs-Cu anode during the self-discharge process.

Table S2. Performance comparison of zinc ion supercapacitors and batteries.

Ref	Cathode	Anode	Electro-lyte	Poten-tial (V)	Gravi-metric capacity (mAh g^{-1})	Specific Power (W/kg)	Specific Energy (Wh/kg)	Areal Capacity (mAh cm^{-2})	Cumula-tive capacity (Ah cm^{-2})	Stabili-ty	N/P ratio
This work	AC 1.26 mg	Cu-Zn	15 M $ZnCl_2$	0-2	246	190 - 1400	210 - 139.7	0.4	19.8	83% 50 k	2.5
1	AC 1mg	NPC Ns/ Cu	2 M $ZnSO_4$	0.2-1.8	70 @5 mA cm^{-2}	1031	71	1	0.8	98% 2k cycles	2.5
2	AC 6 mg cm^{-2}	<u>Zn@ MOF</u>	2 M $ZnSO_4$	0.2-1.8	132 @0.1A/ g	70.0	140.8	0.48	6.19	72% 20k cycles	21
3	AC 0.4-0.8 mg/cm^{-2}	Zn (25 × 10 × 0.25 mm)	[Zn $(bet)_2$][NTf_2]$_2$ (ZbN) (AN)	0-2.5	80 @0.5 A/g	240	118 cathode	0.064	0.64	90% 10 k cycles	>10
4	ANHPC -x, 1 mg cm^{-2}	Zn foil			199.1 @0.5 A/g	41.4	155.2	0.2	13	99% 65k cycles	>10
5	RbPC 0.8 mg	Zn foil	1 M $Zn(CF_3SO_3)_2$	0.2-1.8	216	100	178.2	0.064	0.12	99.8 % 20k cycles	>10
6	MnO_2	Cur-C	/	0.8-1.8	200	/	135 Wh/kg	0.5	0.04	/	>10
7	PANI	10 um Zn	/	/	220 @0.05A /g	900	175	1.8	0.09	68% 80 cycles	
7	PANI	20 um Zn	/	/	77 @10 A/g	10000	110	1.9 @0.2 A/g	0.285	100% 50 cycles	2.7
8	$LiMn_2O_4$	Zn	20 M LiTFSI + 1 M $Zn(TFSI)_2$	~1.7	66	30	180	2.4 @0.2 C	9.6	100% 150 cycles	2.7
9	$K_{0.486}V_2O_5$ @2.5 mg/cm^2	Zn	SCCa 15 M $ZnCl_2$	1.6	400	32.85 (1800)	268.2 (110)	1	2.8	4k cycles	4.1
10	VOPO	Zn-Ti	4M Zn $(OTF)_2$ +0.5 M Me_3Et NOTF		163	0.049	158.6	~0.3	1.62	89% 6k	3

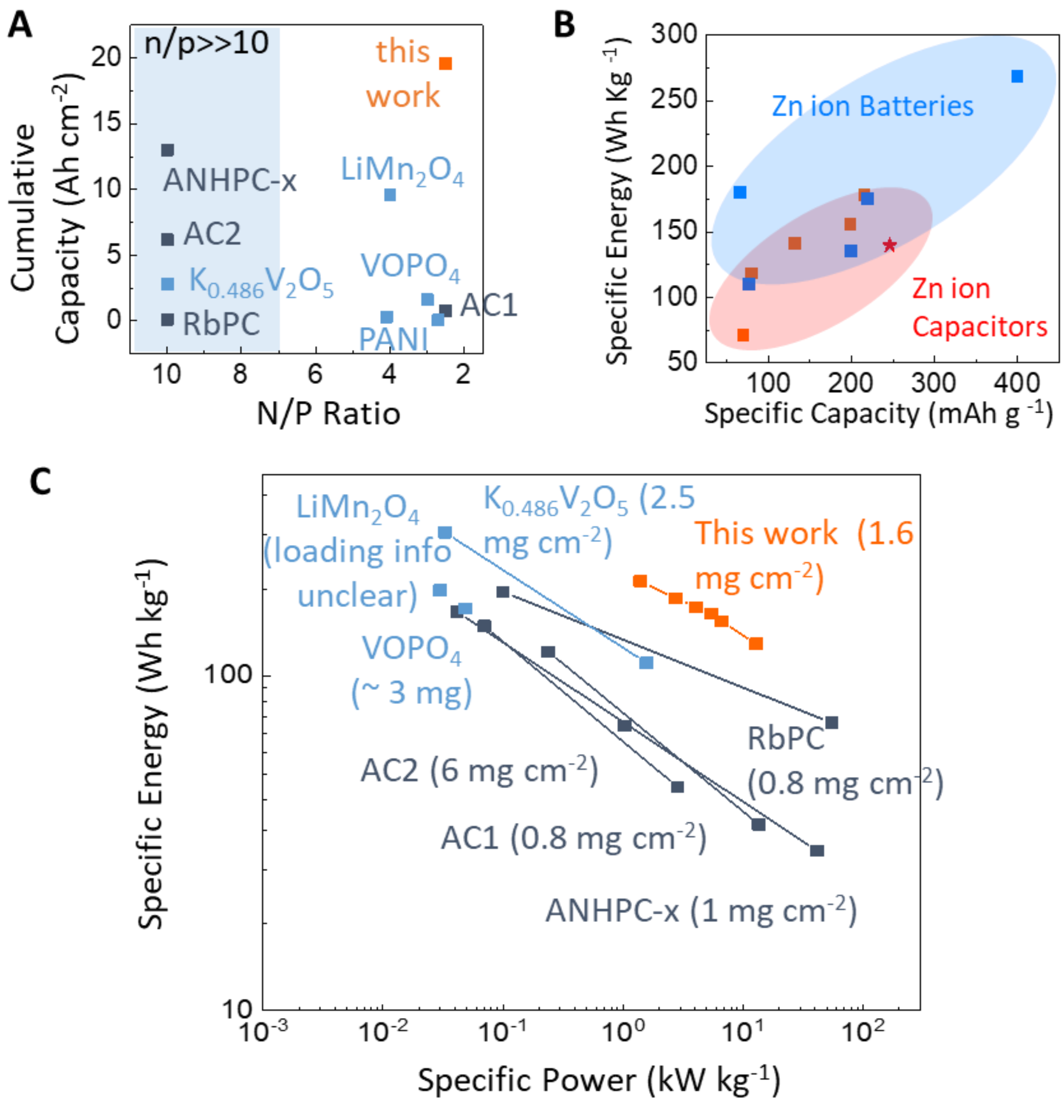

Figure S15. (a) Cumulative capacities of initially anode-free devices. Zn ion batteries (light blue markers): $LiMn_2O_4$,[8] PANI, [7] $K_{0.486}V_2O_5$,[9] $VOPO_4$.[10] Zn ion capacitors (dark blue markers): AC1 (n/p=2.5),[1] AC2 (n/p>10),[2] ANHPC-x,[4] RbPC.[5] (b) Specific capacity and specific energy of zinc ion capacitors and zinc ion batteries. (c) Specific energy and specific power of the devices in part (a). Device with N/P= 2.5: 1.6 mg cm^{-2} active carbon on carbon cloth as cathode; Cu current collector with CuNPs sputtered at 100W as anode current collector; 1.26 mg cm^{-2} active Zn amount; and 200 µL 15 M $ZnCl_2$ as electrolyte.

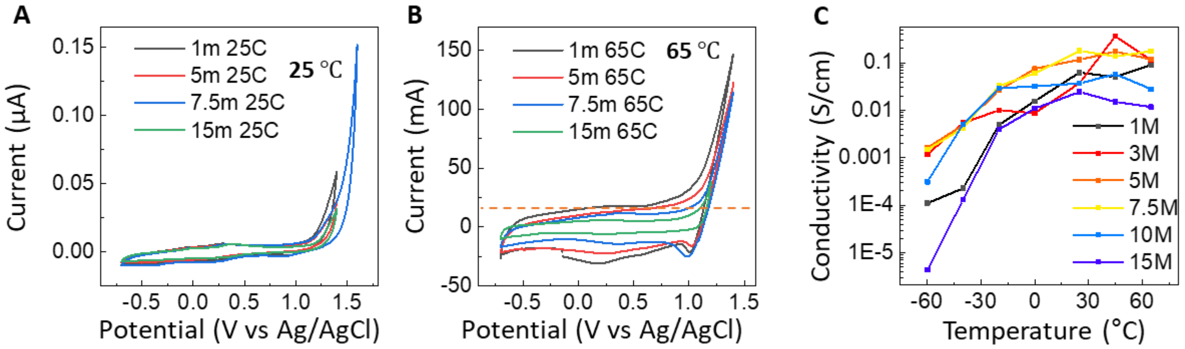

Figure S16. The potential window of water in salt electrolyte (WIS) at different concentration and temperatures.

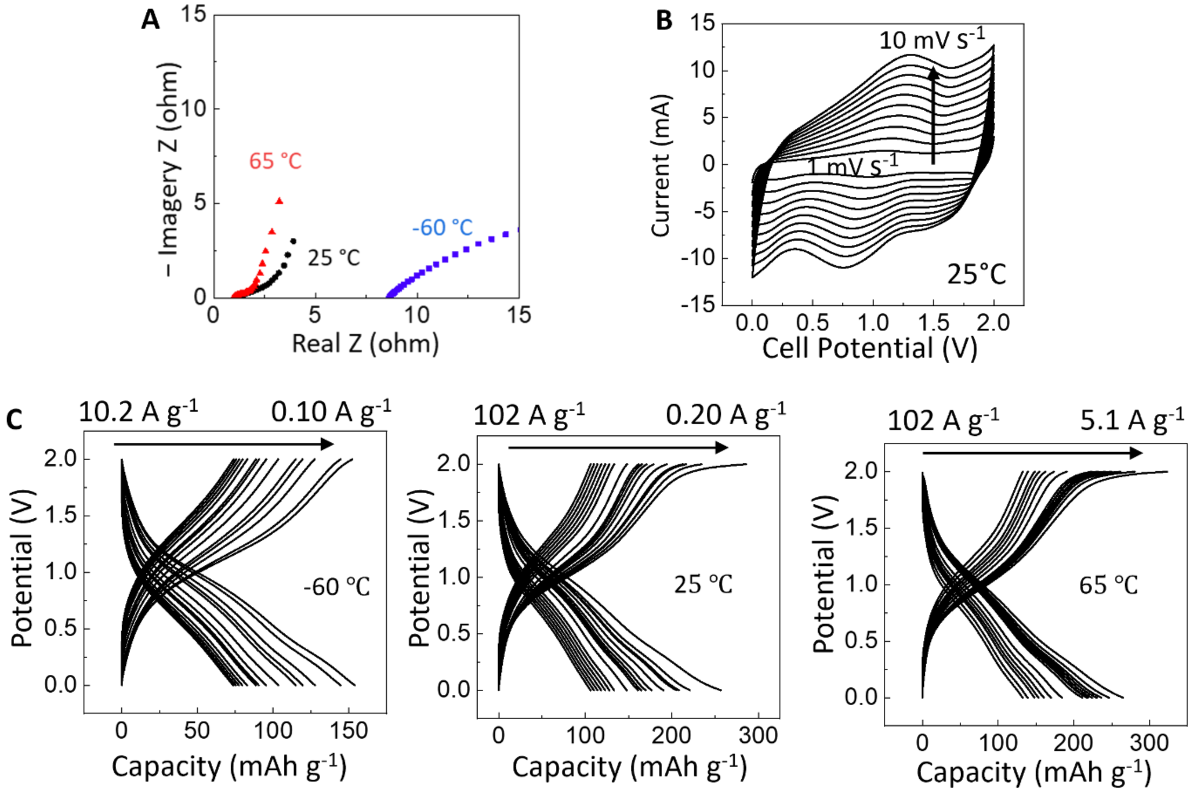

Figure S17. (A) Impedance of the supercapacitor held at 1 V at -60 °C, 25 °C, and 65 °C. (B) CV curves with scan rates of 1 mV/s to 10 mV/s for the zinc ion supercapacitor. (C) Voltage-capacity profiles of the supercapacitor in Figure 5E at various charge/discharge currents and at different temperatures.

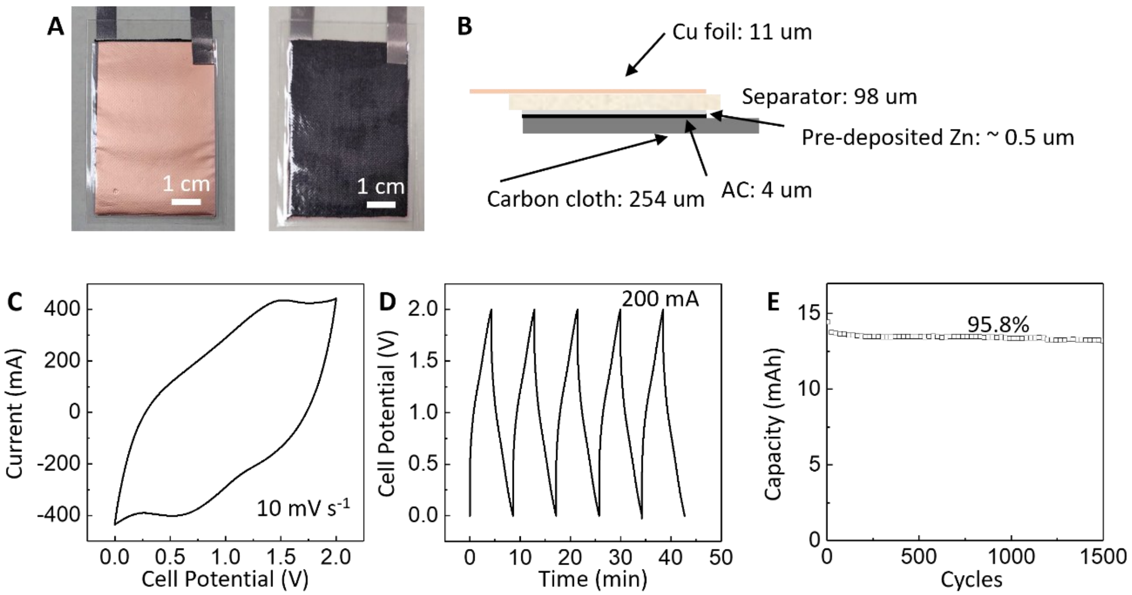

Figure S18. (A) Photos of a large device with a capacitance of 25.79 F and a capacity of 14.3 mAh at a current density of 10 mA cm^{-2}. (B) The structural dimensions of the device. (C) The CV curve of this large device. (D) Galvanostatic charge-discharge characteristics at a current of 200 mA. (E) Cycling stability over 1500 charge-discharge cycles of 2 V.

Table S3. Dimensions and materials loading of the devices measured in this work.

	Components	Dimensions	Notes
Figure 3	Working electrode: Zn on current collector, 0.85 mAh cm^{-2} at 100 % state-of-charge;<br>Counter/Reference Electrode: Zn foil with 100 um thickness;<br>Electrolyte: 200 µL 15 M $ZnCl_2$	Circular carbon-cloth current collector and Zn foil anode are 1 cm in diameter	Glassy carbon as the current collector for the cell.
Figure 4	Cathode: 1.64 mg cm^{-2} AC on carbon cloth;<br>Anode: Zn foil of 100 µm thickness;<br>Separator: Whatman glass microfiber filter (FG/A);<br>Electrolyte: 200 µL 15 M $ZnCl_2$	Circular carbon-cloth current collector and Zn foil anode are 1 cm in diameter	Gravimetric capacity was calculated only with the AC weight, excluding separator, current collectors, and packaging
Figure 5A and 5B	N/P= 4<br>Cathode: 1.12 mg cm^{-2} Cl_2-AC on carbon cloth;<br>Anode: Cu current collector with CuNPs sputtered at 100W. Active Zn amount: 1.41 mg cm^{-2}	Cathode and anode are 1 cm in diameter	Gravimetric capacity was calculated using only zinc and Cl_2-AC weights, excluding separator, current

	N/P=2.5<br>Cathode: 3.26 mg cm^{-2} Cl_2-AC on carbon cloth;<br>Anode: Cu current collector with CuNPs sputtered at 100W. Active Zn amount: 2.54 mg cm^{-2}<br><br>Separator: Whatman glass microfiber filter (FG/A);<br>Electrolyte: 200 µL 15 M $ZnCl_2$		collectors, and packaging
Figure 5C	Cathode: 1.25 mg cm^{-2} Cl_2-AC on carbon cloth;<br>Anode: Cu current collector with CuNPs sputtered at 100W. Active Zn amount: 1.59 mg cm^{-2}<br>Separator: Whatman glass microfiber filter (FG/A);<br>200 µL 7.5 M $ZnCl_2$ electrolyte	Cathode and anode are 1 cm in diameter	Specific energy and specific power was calculated with only Cl_2-AC weight, excluding separator, current collectors, and packaging
Figure 5d	Cathode: 3.5 mg cm^{-2} Cl_2-AC on carbon cloth current;<br>Anode loading:<br>2.69 mg cm^{-2} (N/P = 2.5).<br><br>Separator: 2 layers of cellulose paper;<br>Electrolyte: 2 mL 15 M $ZnCl_2$	Area of 4 cm × 5.5 cm	Ti foil was used as collector tabs, and PET films was used to seal the device
Figure 5e	Cathode: 1.6 mg cm^{-2} Cl_2-AC on carbon cloth current collector;<br>Anode loading:<br>1.26 mg cm^{-2} (N/P = 2.5).<br><br>Separator: Whatman glass microfiber filter (FG/A);<br>200 µL 15 M $ZnCl_2$ electrolyte	Diameters of 1.4 cm for both cathode and anode	Specific energy and specific power was calculated with only Cl_2-AC weights, excluding separator, current collectors, and packaging

Supplementary References

1. Liu, P., Fan, X., Ouyang, B., Huang, Y., Hao, R., Gao, S., Liu, W., and Liu, K. (2022). A Zn ion hybrid capacitor with enhanced energy density for anode-free. J Power Sources *518*, 230740.
2. Wang, Z., Huang, J., Guo, Z., Dong, X., Liu, Y., Wang, Y., and Xia, Y. (2019). A metal-organic framework host for highly reversible dendrite-free zinc metal anodes. Joule *3*, 1289–1300.
3. Chen, P., Sun, X., Pietsch, T., Plietker, B., Brunner, E., and Ruck, M. (2023). Electrolyte for High-Energy-and Power-Density Zinc Batteries and Ion Capacitors. Advanced Materials *35*, 2207131.

4. Wang, L., Peng, M., Chen, J., Hu, T., Yuan, K., and Chen, Y. (2022). Eliminating the Micropore Confinement Effect of Carbonaceous Electrodes for Promoting Zn-Ion Storage Capability. Advanced Materials *34*, 2203744.
5. Wang, L., Peng, M., Chen, J., Tang, X., Li, L., Hu, T., Yuan, K., and Chen, Y. (2022). High energy and power zinc ion capacitors: a dual-ion adsorption and reversible chemical adsorption coupling mechanism. ACS Nano *16*, 2877–2888.
6. Zhu, Y., Cui, Y., and Alshareef, H.N. (2021). An anode-free Zn–MnO2 battery. Nano Lett *21*, 1446–1453.
7. Cheng, Y., Jiao, Y., and Wu, P. (2023). Manipulating Zn 002 deposition plane with zirconium ion crosslinked hydrogel electrolyte toward dendrite free Zn metal anodes. Energy Environ Sci *16*, 4561–4571.
8. Wang, F., Borodin, O., Gao, T., Fan, X., Sun, W., Han, F., Faraone, A., Dura, J.A., Xu, K., and Wang, C. (2018). Highly reversible zinc metal anode for aqueous batteries. Nat Mater *17*, 543–549.
9. Li, L., Liu, S., Liu, W., Ba, D., Liu, W., Gui, Q., Chen, Y., Hu, Z., Li, Y., and Liu, J. (2020). Electrolyte Concentration Regulation Boosting Zinc Storage Stability of High-Capacity K0.486V2O5 Cathode for Bendable Quasi-Solid-State Zinc Ion Batteries. Nanomicro Lett *13*, 34. https://doi.org/10.1007/s40820-020-00554-7.
10. Cao, L., Li, D., Pollard, T., Deng, T., Zhang, B., Yang, C., Chen, L., Vatamanu, J., Hu, E., Hourwitz, M.J., et al. (2021). Fluorinated interphase enables reversible aqueous zinc battery chemistries. Nat Nanotechnol *16*, 902–910. https://doi.org/10.1038/s41565-021-00905-4.
